# A Treatise on the Venereal Disease

**Published:** 1854-01

**Authors:** 


### EDITORIAL.

*A Treatise on the Venereal Disease.* By John Hunter, F. R. S., with copious additions by Phillip Ricord, Surgeon of the Hospital du Midi, Paris, etc., edited with notes by Freeman J. Bumstead, M. D., New York. Blanchard & Lea, Philadelphia, 1853.

SIXTEEN years have elapsed since the publication of the American edition of Hunter, on the Venereal disease, with Babbington's notes: during this period much has been added to our knowledge by Ricord, of Paris, and Acton of London. In the work before us we have the extensive experience and critical observation of the best syphilographists of the day added to the sagacity of the father of modern surgery—the wisdom of Hippocrates, and the polished expression of Celsus. Although many of the views of Hunter are now obsolete, he has nevertheless left us a splendid monument of untiring industry and speculative genius.

On comparing the first American with the present edition, we find the original method has been strictly regarded: the annotations of Babbington, the additions of Ricord and the American editor have been judiciously inserted in the respective chapters and subdivisions. In part IV. chap. I., we have the fullest exposition of M. Ricord's views; next in part VI., chapt. I., and in part III. chap. XIV. and XV. he has given us valuable matter on gonorrheal opthalmia, and gonorrheal rheumatism. Much could not be expected from the American editor, save a clear translation and proper arrangement of his material; the few notes he has added show his task has been performed with judgment. Those opinions of Hunter, which have stood the test of nearly a century, need but a passing notice, and will be cited, chiefly, to corroborate or refute the authority of his successors.

Ricord substantially denies the proposition of Hunter and Babbington, that gonorrhœa may be communicated to the woman before it has appeared in the man. Mr. Babbington extends his peculiar views to chancre, and says that infection may take place from a simple chancrous thickening, although no ulceration whatever is present. Ricord explains this in the following manner. "Chancres may be developed on a follicle in the cellular tissue or on the superficial lymphatic vessels, and be accompanied by surrounding induration which encloses them in a sort of shell or cyst. Under these circumstances they may appear to be simple indurations without suppuration, whilst during sexual intercourse the walls of the abscess gape. The pus escapes and communicates the disease, which is wrongfully attributed to a consecutive ulceration." We think this reasoning ingenious, if not demonstrably correct.

The Hunterian doctrine, that the poison of syphilis and gonorrhœa are the same, has been refuted. Although we have the valuable monographs of Tode, Hernaudez, Swediaur, Bell and Duncan, no clear summary of facts had been given until the publication of Ricord's experiments. The following are the results of the experience of more than twenty years :

I. *Whenever muco-pus is taken from a surface free from ulcerations—no matter what the previous symptoms may have been, the seat of the disease, its duration or its intensity—the results of its artificial inoculation are negative, in whatever tissue the experiment is made.*

II. *The existence of a mere ulceration, without distinction of kind upon mucous surfaces affected with a running, is not sufficient to produce chancre.*

III. *When I take muco-pus or pure pus from a mucous surface that I have not previously explored, and unable to obtain positive result by inoculation, I am forced to infer the existence of a concealed chancre.*

On this interesting subject the editor quotes from the recent work of M. Vidal, well known as one of Ricord's bitterest opponents. He says, "the *most frequent* and *effective cause* of gonorrhœa is virulent pus. * * * "It is difficult to distinguish the two, but if you can satisfy yourself that there was true incubation in the case, and if the discharge tends to a chronic state, you will incline to believe it specific, and *vice versa;* and if syphilitic symptoms afterwards appear, the diagnosis will be plain." Hunter, in writing on the difficulty of distinguishing the virulent from the simple gonorrhœa, doubts very much the possibility of a person getting a fresh gonorrhœa while he has that disease; this is fallacious, for experience teaches us that repeated attacks cause a proclivity to infection, therefore, *a fortiorii*, we are right in assuming that an individual with gonorrhœa may contract the disease afresh.

Different men are differently affected, one may cohabit with one particular woman who is diseased and escape, another may shortly follow and contract a clap. We have seen several cases of men *diseased* by wives who were perfectly chaste, but suffering from vaginal irritation, increased during *coitus*. In such individuals there is unusual susceptibility of the whole mucous system, and the slightest atmospheric change is sufficient to produce *coryza* or *bronchitis*. One case which came under our notice, recently could not be distinguished in its inception from the most aggravated form of specific disease; its disappearance, however, in a week proved its benign character.

Of the cure of gonorrhœa: Hunter tells us to have in view the possibility of some of the matter being absorbed, and afterwards appearing in the form of Lues venerea. It would be supererogatory to say that no treatment directed to such an end could be more erroneous, and should any surgeon at the present day advocate such a course, he would meet with censure if not ridicule.

Ricord gives the result of his experience in the treatment of gonorrhœa, which he considers definitively local in its character. "The pretended danger of a rapid cure or of driving in the discharge is imaginary, and the contrary proposition may be proved, viz.: the more rapid the cure, the more speedy security from complications."

He divides his abortive treatment into the *direct* and *indirect;* under the former head he places the nitrate of silver; under the latter copaiba and leeches. He uses a quantity of the nitrate which our experience convinces us is entirely too small. We prefer Carmichæl's solution, ten grains to the ounce, and have used as high as thirty, with less pain and inconvenience to the patient than two or three grains. There are individuals however of irritable constitution who cannot bear this active treatment, it is therefore obligatory on the surgeon to exercise judgment and discrimination.

Neither Ricord nor the editor mention the treatment of gonorrhœa by chloride of zinc injections, in proportion of one grain of the salt to an ounce of water. When the disease has passed to the second stage, Ricord uses copaiba and cubebs in combination, but does not accord to the latter all the virtues ascribed to it by Sir Astley Cooper; he agrees with Cullerier that there is something peculiar in the action of these agents on the *diseased* urethral membrane and that gonorrhœal discharges from the lower part of the rectum are not affected by them.

There are individuals on whom the usual remedies have little or no effect—the disease running on mitigated, but unchecked. In these we have usually found, on passing a bougie, a tender spot in the prostatic portion of the urethra. Our treatment of these cases is cauterization and the internal administration of the *Diosma crenato* (Buchu), combined with the tincture of the sesquichloride of iron. We fully agree with Ricord that the doses of copaiba usually given are entirely too small; we believe, too, that it is useless to protract its use beyond a fortnight or three weeks.

The editor informs us that it is exeedingly difficult to make the stomach tolerate the chief remedial agent—copaiba; he advises the formula of Velpeau, Vidal, Maissoneuve; had he consulted the London Lancet for Nov. 6, 1852, he would have found a description of the admirable *copahine mege capsules*, by M. Jozeau.

In the treatment of chordee Ricord uses pills of camphor and opium; when these are not successful he advises compression of the prepuce in front of the gland, first proposed by Dr. Sistock. Nothing is said about a medicine which has recently been used with much success in this troublesome affection, we refer to Lupulin; this should be given in doses of ten to fifteen grains in a little syrup, it possesses the advantage over other anaphrodisiacs that it causes no headache or constipation, and does not give rise to nervousness or any other unpleasant consequence.

The editor has appended to Ricord's treatment of epididymitis, a description of the operation of Velpeau of incising the tunica vaginalis, and that of Petit revived by Vidal of incising the body of the testicle. Vidal says, "in spite of the prejudices against this operation, he has performed it four hundred times without any bad result, and that it is always harmless if the incisions be confined to three-fourths of an inch in length."

In part III. chapter II., we have the treatment of strictures of the urethral canal. The different methods are taken up *seriatim.* Hunter informs us that the time a bougie should remain in the passage must be determined by the feelings of the patient; for it should never give pain if possible.

We have seen but few cases in which the bougie could not be used if properly handled; an injection of cold water, if the stricture be not very tight, with tincture of hyosciamus or tincture of opium into the rectum will reduce the morbid irritability of the parts. The different plans of dilatation of Mayor, Ducamp, Chrestien, Lallemand, and Velpeau are cursorily reviewed by Ricord: he prefers, however, Hunter's method of gradual dilatation. We must be guided in our treatment by the condition of the patient—it is not easy to lay down any invariable rule for the use of the bougie. In several obstinate cases of old standing strictures, we have succeeded in effecting a satisfactory cure by the following method: We take a metallic bougie of a size that will pass readily, and allow it to remain with the penis in a depending position until it falls out; then another of a larger size is introduced, and allowed to remain until its weight overcomes the contractile power of the stricture; this is repeated at first every day, after a while twice a day, until the largest size will pass. Combined with this instrumental treatment, we use as a resolvent the *unguentum hydragyri fortior*, rubbed up with camphor, and applied with friction along the urethra externally, while the bougie remains within the canal. After the instrument has been introduced two or three times, there will be aggravation of the discharge which before *mucoid* becomes *muco-purulent;* at the same time there may be chordee—these, however, will pass off in the progress of the cure.

The use of caustic, which is so highly lauded by Whately, is recommended by Hunter only in those cases where the stricture cannot be passed; in its application Ricord prefers Lallemaud's instrument; his editor should have noticed the improvement on it by Thompson, of London, which we think has greater merit than the original. In the treatment of strictures by incision, Ricord rejects the instruments of Amussat, Segalas, Leroy d'Etiole, Tanchou, Guillon, Reybard, etc., all of which have some merit, and substitutes one of his own, which is figured in plate III. Finally, Ricord gives as a succinct account of Mr. Syme's operation by external incision. Our space will not admit us to consider at length this subject, which has brought upon its originator the thunders of the London school. We think it uncertain, and only to be performed in extreme cases of retention of urine. For interesting information on this subject we refer our readers to Mr. Syme's papers in the Edinburgh Medical and Surgical Journal, and to a paper by Mr. Guthrie in the London Laucet for June, 1851, and one by Mr. Simon in Ranking's Abstract, Vol. VIII.

Ricord has contributed valuable additions on the cause, course, and treatment of gonorrhœal opthalmia, and gonorrheal rheumatism; he divides the opthalmic disease into direct and sympathetic. In speaking of its treatment he has overlooked Tyrell's plan of incisions of the cornea, and leeches around the eye. Gonorrhœal rheumatism was first recognized by Swediaur; it is frequently caused by the too free use of antiblenuorhagics; repercussion; it is rare during the first week. The predisposing causes are cold and moisture. The *femoro tibiol* articulation is most frequently attacked in men, and, according to Cloquet and the *coxo-femoral* in women. The affected joint swells from effusion into the synorial cavity, in other words it is a subacute *hydrathus*. The remedies are the same as for common rheumatism.

There is an affection of the corpus cavernosum, the result of gonorrhœa, which was first described by Henry James Johnson, of London, and is not noticed by the editor; after observing this omission, we cannot give him as much credit for research as we had wished.

In part IV. chapter I., we have the subject of chancre discussed. Ricord in his addition asserts what he has before taught, that there can be no constitutional syphilis without a primary sore, except through hereditary transmission—that chancres, wherever situated, furnish specific and contagious pus. The results of artificial inoculation are more certain than the effects of ordinary contagion; and virulent pus which has been preserved a long time in tubes, may be inoculated like vaccine virus. Ricord denies what Hunter asserts, that there is a period of incubation for chancres; he lays down the proposition that *induration is a certain proof that the system is contaminated.* Our space will not allow us to discuss the mooted question of the unity or plurality of the syphilitic virus. We believe in the existence of one virus, which may be modified in the constitution of different individuals. Ricord, page 290, lays down the following unexceptionable rules: "*There are two things to be considered in the cure of a chancre, first—the cicatrization or disappearance of the ulcer; secondly—the state of the tissues beneath it. A cure is really affected only when the induration disappears. An indurated chancre which cicatrizes very rapidly will be followed by constitutional symptoms as well as one which continues a longer time; with it a more indurated chancre, which is as long or even longer in getting well than the former, will cause nothing of the kind. Induration being requisite for the production of the constitutional phenomena of syphilis, no matter how long the primary ulcer lasts.*

In page 292, we are informed by Ricord that secondary symptoms have rarely if ever followed chancres which were destroyed before the fifth or sixth day after the infecting coitus. He refutes the charge that cauterisation favors the development of buboes.

The use of mercury in chancre is discussed in page 315, et seq. —A more rational course of treatment has supplanted the empirical mode of the early syphilographists. This subject has caused much controversy between the army and the civil surgeons of Great Britain; the former beginning with Dr. Hennen, have almost exclusively rejected mercurials; the latter use them in nearly every case. Mr. Rose, surgeon to the Guards in London, treated a large * number of his men on the non-mercurial plan with decided success, only three requiring the old treatment. On the other hand Lawrence, Brodie, Cooper, Abernethy, Delpech, etc., deem mercury indispensable. Ricord and the French School, generally, are opposed to mercury for simple chancre. We think there is an intermediate course which the judgment of a skilfull surgeon will lead him to follow. He should remember the advice given by Phœbus to Phœton—"If you ascend too high, you will burn the heavenly mansions; if you descend too low, you will burn the earth to ashes; do not drive to the right, you will there meet with the constellation of the serpent; avoid going too much to the left, you will there fall in contact with that of the altar; keep in the middle."

In military practice our experience enables us to say that the non-mercurial treatment is more suitable than in civil practice. Men are more under control and cleanliness can be better enforced. We have seen simple chancres become phagedenic under mercury, and we believe that buboes will oftener follow chancre when treated with it than without it—there is, however, the indurated chancre which always requires it.

Ricord, in opposition to Hunter, who makes but one kind of bubo, makes seven. We have space to notice but a few of these. *Primary syphilitic*, this has always a chancre for its antecedent. *Non-consecutive*, whose only antecedent is sexual intercourse without any primary symptoms: inoculable pus cannot be obtained from it. *Benignant*, which follows gonorrhœa. *Constitutional or Secondary*, is identical in its nature with other manifestations of constitutional syphilis, as swellings of the cervical ganglia, etc.

In the abortive treatment of buboes, Ricord advises compression; this we have found can rarely be borne. Malapert proposed cauterization of the superimposed integument, we believe, this is seldom successful. If a bubo is indolent, we prefer the strong mercurical ointment, combined with camphor, rubbed in twice daily for three consecutive days.

* Having mislaid our notes we cannot give the figures.

In opening a bubo, we think caustic is to be preferred, unless it is quite small; it then heals from the bottom, and there is no superfluous skin left.

In the division of syphilitic symptoms Hunter and Ricord agree. The primary ulcer rarely lasts long enough for tertiary symptoms to be developed; and the treatment which modifies the secondary symptoms and keeps them under its influence so long as its action lasts may not have the least prophylactic effect on the tertiary symptoms.

We now come to a most interesting branch of syphilography—cutaneous affections. Hunter divided them into *tubercle*, seated in the sebaceous glands; it first appears as a small hard substance like a pea, the inflammation quickly extends from beneath to the surface, sometimes it ulcerates and acquires the characteristic brownish tint. *Lichen*, consisting of small acuminated pimples, some of which imperfectly suppurate.

*Psoriasis and Lepra.* The scaly eruptions are very common in venereal disease; there is but little difference between them when arising from other causes.

*Rupia.* This begins on the surface of the cutis and sometimes resembles ulcerating tubercles.

Modern Dematologists divide syphilitic cutaneous disease into *exanthematous*, *papular*, *squamous*, *vesicular*, *pustular, and ulcerous.* Syphilitic eruptions occur in those, who have taken no mercury for an indurated chancre. The parts of the body affected are in the following order—on the trunk and extremities, about the genital organs, scalp, face, sole of the foot, and palm of the hand—and the external meatus auditorius. The peculiar form of the primary ulcer has no influence on the value of the secondary acception. Ricord goes so far as to say there is not a single variety of cutaneous affection which may not be produced by syphilitic virus.

Among the heriditary diseases none so justly deserve the title as syphilis; while yet the child is in embryo, it may become infected through the constitution of the mother; it may, too, receive syphilitic infection from the sire. "If either parent, or both have the symptoms of constitutional syphilis, whether developed or undeveloped, the disease will show itself on the infant a few weeks after birth. There is a disease which Paul Dubois has recently noticed as due to constitutional syphilis in infants—*pemphigus*; this is often difficult to distinguish from the ordinary disease by the same name.

The treatment of infantile syphilis, requires considerable care. We prefer that of Brodie to all others. He advises mercurial ointment spread over a flannel roller and wound around the child's body: the restlessness and kicking of the child causes irritation of cuticle and thus facilitates the absorption of the mercury. When given in this way, it does not either gripe or purge, nor does it cause sore gums.

Under the head of tertiary syphilis, Ricord places syphilitic *sarcocele* so well described by Bell, Cooper and Dapuytren, and denied by Hunter. Ricord gives it the name of *albuginitis*. He considers it one of the first symptoms of tertiary syphilis. It may appear after several months, and it may be postponed for years. This disease is stated in the tunica albuginia of the testicle: the epididymis and vas deferens are not often involved unless the patient has had gonorrhœal *epididymitis*. The slightest exciting cause, as a blow or strain, in an individual suffering from constitutional syphilis, may prove an exciting cause. Sometimes this affection of the testicle begins with atrophy; indeed, Vidal thinks the gland may become atrophied without any previous lesion. Constitutional syphilis alone being both the predisposing and the exciting cause. Ricord says the mistake of this assertion may be accounted for by the knowledge that partial *albuginitis* may occur without becoming so large, or so painful as to excite the attention of the patient. Ricord says, "*it may be laid down as a rule, that whenever syphilis alone acts on the testicle, suppuration never occurs; tubercular tumours of the scrotum, like other syphititic tubercles of the cellular tissue can alone suppurate.*" We may here advert to the animosity existing between Ricord and Vidal, and would advise our readers to take *cum grano salis*, whatever reciprocal contradiction they may make.

Ricord gives some judicious rules for administering mercury. "Give it internally when the digestive organs are in good condition; if the stomach will not tolerate it, use inunction; if both stomach and skin are irritable, we should combine the two methods. If the patient cannot be brought under its influence by these methods, we should use fumigation. If after a week the patient's constitution shows no sign of favorable change, the dose should be increased. Mercury has been found in nearly all the tissues after death; M. Regnand, of Toulon, found it in the brain, M. Grassi, of the Hospital du Midi, found it in the suppurating surface of the left anterior lobe of the cerebrum; M. Chevalier says it does not pass into the milk of nurses, while Barruel found it in the breasts of a female who died of puerperal peritonitis.

The editor has appended to Ricord's observations on tertiary syphilis, an abstract from a Thesis recently published in Paris, on syphilitic disease of the lungs. The author, M. Lagneau, has collected fifty-three cases in which syphilis was the "unequivocal" cause of the existing changes. M. M. Depaul, Dubois and Gubler have observed abscesses of the thymus gland, coincident with pemphigus, *neo natorum*, and syphilitic disease of the liver, consisting in the exaltation of fibro-plastic matter, the result of pre-existing inflammation.

It is well known that M. Ricord is a strenuous advocate for the use of Iodine in tertiary syphilis, he has, however, become more exclusive than formerly. That it is a valuable medicine we will not deny, but we would ask our readers who may peruse this work to keep before them the Horatian maxim, *Nullius addicta jurare in verba magistri.* Absolutism in medicine is the offspring of French institutions; eclecticism is the child of our own soil. We have seen those who were utterly confounded, when iodine failed, because they had learned no other treatment. There is a medicine which M. Ricord does not notice; we refer to the decoction of Zittm[illegible]n We have used this extensively in a large elumosynary ins[illegible]itut[illegible]on, both as a remedial agent, *per se*, and as an adjuvant to the iodine treatment, and we are satisfied with its *autrophic* powers.

The following is a *resume* of this very intractable form of syphilis. Iodide of Pottassium beginning with doses of two scruples a day, in decoction of hops or sarsaparilla, its effects on the dejective organs, and its producing a kind of ptylaism should not be overlooked. *

When the tertiary and secondary stages run into each other, a condition which is readily recognised by the presence of deep tubercles of the skin, and mucous membranes, the protoiodide of mercury is to be given. In the squamous eruptions use an ointment of tar and cerate in the proportion of one part of the former to three of the latter.

In part VI., chap. V., Ricord has given some judicious advice on the *prophylaxis* of the venereal disease. As it is more particularly adapted to the meridian of Paris, we will not transcribe it: but would refer those, whose libidinous propensities are uncontrollable to it for perusal. We are glad to see a book containing so much varied and valuable information within reach of all medical men, and we hope they will avail themselves of its useful precepts. The translator of Ricord's additions has done his duty well, and given us for pure Gallic, the vigorous Anglo Saxon. The typography is respectable and the lithographic plates are excellent.

For sale by Keen & Bro's. W. H. T

* Atrophy of the breast and testics has been said by Mr. Hill (Edinburgh Medical Journal Vol. XXV.,) to have resulted from its use. We believe the charge unfounded.

